# Synergistic Effects of Tomato and Soy in Gluten‐Free Noodles: Unveiling Antioxidant, Physicochemical, and Sensory Properties

**DOI:** 10.1002/fsn3.71944

**Published:** 2026-05-23

**Authors:** Mitu Rani Sarker, Md. Jaynal Abedin, Abu Tareq Mohammad Abdullah, Tasnim Farzana

**Affiliations:** ^1^ Institute of Food Science and Technology (IFST) Bangladesh Council of Scientific and Industrial Research (BCSIR) Dhaka Bangladesh

**Keywords:** antioxidants, gluten‐free, noodles, nutrition, sensory, tomato

## Abstract

The growing prevalence of gluten‐related disorders illustrates the importance of healthier and functional gluten‐free foods. Tomatoes and soybeans are rich in antioxidants, phytochemicals, and important macro‐ and micronutrients. This study aimed to evaluate the impact of incorporating different proportions of tomato powder (TP) (5%–20%) and defatted soybean flour (DSF) (10%) in gluten‐free noodles on their antioxidant potential, nutritional composition, physicochemical properties, and sensory attributes. A significant increase (*p* < 0.05) in antioxidant potential of the noodles was observed, reflected by elevated phenolic (140.32 ± 1.86 to 250.05 ± 1.88 mg GAE/100 g), flavonoid (71.64 ± 0.49 to 158.28 ± 0.08 mg QE/100 g), total antioxidant (147.88 ± 2.29 to 392.32 ± 2.08 mg AAE/100 g), DPPH activity (21.7% ± 0.49% to 52.12% ± 0.76%) and FRAP (94.96 ± 1.28 to 205.30 ± 1.78 mg AAE/100 g) values. Notable enhancements in dietary fiber (17.94 ± 0.17 to 24.50 ± 0.04 g/100 g), protein (16.87 ± 0.07 to 17.21 ± 0.04 g/100 g), and minerals like Ca, Mg, and Mn were noticed. The water absorption capacity and cooking yield of the noodles have significantly improved (*p* < 0.05). However, the texture analysis revealed a softer texture without any significant (*p <* 0.05) changes in cohesiveness, springiness, and adhesiveness. Sensory evaluations indicated that the inclusion of 15% TP and 10% DSF yielded optimal sensory attributes regarding taste, flavor, and overall acceptability. These findings suggest that TP and DSF can be effectively used to improve the nutritional, functional, and sensory attributes of noodles while providing an excellent gluten‐free alternative with potential health benefits and consumer acceptability.

## Graphical abstract

Gluten‐free noodles made from foxtail millet flour and enhanced with defatted soy flour and tomato powder increased the goods' nutritional, antioxidant, cooking, color, texture, and sensory attributes. Protein, dietary fiber, potassium, calcium, manganese, and magnesium levels were higher in the evolved noodles, whereas fat, carbohydrate, and calorie values were lower. Antioxidant activities such as DPPH radical scavenging activity, FRAP assay, total phenolic content (TPC), total flavonoid content (TFC), and total antioxidant capacity (TAC) were all significantly increased. Cooking quality enhanced with increased water absorption and yield, as well as reduced cooking loss. Color parameters a*, b*, browning index (BI), Δ*E*, and chroma increased whereas L*, hue angle, and whiteness index (WI) declined. The texture profile study revealed lower hardness, gumminess, adhesiveness, and cohesiveness. Among all formulations, N3 noodles exhibited the highest sensory acceptance.

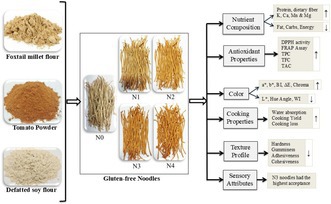

## 1 Introduction

Gluten‐free diet has gained significant attention due to the growing prevalence of gluten‐related disorders, which are estimated to impact approximately 5% of people worldwide (Bascunan et al. 2018). A range of conditions, collectively referred to as gluten‐related disorders (GRDs), can arise from the consumption of gluten proteins found in wheat, barley, and rye; these include celiac disease (which has a global annual increase in cases of 7.5% approximately), dermatitis herpetiformis, non‐celiac gluten sensitivity, gluten ataxia, and allergies (Singh et al. 2018; Capriles et al. 2023). To effectively prevent symptoms and complications associated with celiac disease, it is essential to strictly adhere to gluten‐free diets. However, this can be quite challenging due to widespread issues with the quality of many gluten‐free products, especially regarding their nutritional value, texture, flavor, mouthfeel, consistency, and overall consumer acceptance (Mazzola et al. 2024; Singla et al. 2024). Research has indicated that gluten‐free diets often have elevated levels of saturated fatty acids and cholesterol, as well as higher glycemic indices, which may be associated with increased insulin resistance, risk of cardiovascular disease, weight gain, and metabolic syndrome (Mazzola et al. 2024). Optimizing storage stability is another challenge, with studies showing that packaging type, temperature, and moisture/fat migration strongly influence sensory and physicochemical quality in GF biscuits, where sensory factors contribute most to overall acceptability (Singh and Kumar 2019). These findings highlight the significant need for advancements in nutritional benefits, safety, and desirable sensory attributes of gluten free food products.

Foxtail millet ( *Setaria italica* *L*.), one of the earliest cultivated crops in Asia and Africa, is abundant in proteins (gluten‐free), dietary fiber, minerals, polyphenolic compounds, and possesses low‐glycemic index (anti‐diabetic), hypolipidemic, hypocholesterolemic, nephroprotective and anti‐oxidant properties (Verma et al. 2020; Dhiman et al. 2024). It emerges as a beneficial dietary choice for those dealing with inflammatory bowel disease (Gunalan et al. 2024) Research indicates that millet flours function as an excellent ingredient in developing innovative gluten‐free foods such as biscuits, bread, noodles, soup etc. that are rich in fiber, phenolic compounds, and minerals while having a lower glycemic index than the staple food made of other flours and rice (Arora et al. 2023; Asrani et al. 2023). Gluten‐free formulations using foxtail millet, amaranth, and copra meal have demonstrated considerable fat and sugar reductions while maintaining overall quality (Singh and Kumar 2018). Additionally, some processing treatments such as soaking, germination, microwave processing and fermentation have been shown to significantly enhance millet quality by increasing protein and fiber contents while reducing antinutritional factors such as tannins and phytic acid (Ajay Singh et al. 2017).

Tomatoes ( *Solanum lycopersicum* ) serve as a remarkable source of antioxidants, carotenoids, dietary fiber, along with essential vitamins and minerals, notably vitamin C, vitamin A (as beta‐carotene), vitamin K, potassium, and folate (Collins et al. 2022). Lycopene is a prominent component of tomatoes, recognized for its anti‐inflammatory properties and its role in lowering the risk of chronic diseases, including cardiovascular conditions and specific types of cancer (Khan et al. 2021). Due to the remarkable nutrient composition and ability to enhance sensory qualities, tomato and its byproduct have emerged as a compelling ingredient for creating healthier and more nutritionally balanced food products such as bread, cookies, crackers, and snacks (Silva et al. 2023; Ray et al. 2016). Therefore, their incorporation into gluten‐free noodles holds strong potential for enhancing nutritional and sensory quality.

Soybeans ( *Glycine max* ) are a nutrient‐dense legume with a high protein content and vital minerals, notably divalent cations such as Ca^2+^, Fe^2+^, and Mg^2+^ along with the monovalent cation K^+^ which have health benefits, especially to females (Kumari et al. 2014). They have a lot of bioactive substances, like isoflavones, saponins, and phytosterols, which are antioxidants and can help with illnesses which are long‐term such as diabetes, cancer, heart disease, and chronic kidney disease (O'Keefe et al. 2015). Defatted soybean flour, a valuable byproduct of soybean processing, is a rich source of protein (48.9%), containing necessary amino acids (Mashayekh et al. 2008). It is progressively being adopted in gluten‐free products owing to its beneficial attributes in enhancing the overall quality and nutritional profile of gluten‐free products. Additionally, it contributes to matrix structuring, texture, and moisture retention in gluten‐free formulations, rendering it an optimal selection for the production of gluten‐free noodles, bread, and various pasta items (Egea et al. 2023).

Noodles often have inadequate nutritional quality, such as low protein, fiber, and bioactive compounds and high refined starches, etc., which are associated with high health risks with persistent intake. Gluten‐free noodles made only of foxtail millet flour often might have imbalanced nutritional quality, unsatisfactory sensory characteristics, and poor physical properties. Addressing these challenges necessitates integrated ingredient approaches. Tomato powder and defatted soybean flour present a complementary solution. While both ingredients have been explored separately in gluten‐free or fortified food products, there has yet to be a comprehensive study that systematically investigates their combined effect on nutritional composition, antioxidant potential, cooking qualities, and sensory performance in gluten‐free noodles. This research addresses the existing gap by proposing that the targeted addition of tomato powder and defatted soybean flour into gluten‐free noodles will improve their nutritional, antioxidant, and sensory properties while also maintaining desirable texture and cooking qualities, providing a functional and healthy option.

## 2 Materials and Methods

### 2.1 Reagents and Chemicals

Methanol, Sulfuric acid, Sodium carbonate, Sodium hydroxide, Aluminum nitrate, Acetic acid, Acetone, Potassium acetate, and Ammonium molybdate were provided by Merck (Germany). DPPH (2,2‐diphenyl‐1‐picryl‐hydrazyl), Folin–Ciocalteu's reagent, Quercetin (Q), Gallic acid (GA), Ascorbic acid (AA), Carboxymethylcellulose (CMC), and Xanthan gum were supplied by Sigma‐Aldrich (USA).

### 2.2 Raw Materials

Foxtail millet (variety: BARI Kaon‐2), tomatoes (variety: BARI Tomato‐14) were collected from the Bangladesh Agricultural Research Institute (BARI) and defatted soy flour (DSF) was procured from the local market in Dhaka, Bangladesh. All the additives were collected from a nearby market and confirmed to be of food‐grade quality.

#### 2.2.1 Preparation of Foxtail Millet Flour (FMF)

Foxtail millet grains were first cleaned and subjected to a soaking treatment (1:3 w/v for 8 h at room temperature) to reduce inherent antinutritional factors. After washing, the foxtail millet grain was dried at 60°C for 8 h in a drying oven (D‐91126, Memmert, Germany). Subsequently, dried grains were finely processed into flour by using an electric grinder (MX‐AC400, 1000 W, Panasonic, Japan). To ensure uniform particle size, the flour was sieved through a 250 μm mesh. The sieving and grinding process was repeated multiple times until a consistent fine powder was obtained. The obtained foxtail millet flour (FMF) was stored at ambient temperature in sealed containers.

#### 2.2.2 Preparation of Tomato Powder (TP)

Following a thorough cleaning, the tomatoes were cut into slices. The tomato slice was dried for around 8 h at 60°C in an oven until a target moisture content of approximately 8%–10% was achieved, as determined by periodic weighing (Memmert D‐91126, Memmert, Germany). After drying, fine (250 μm) tomato powder (TP) was produced using a grinder and subsequently kept in an airtight polythene bag (food‐grade) in a refrigerator.

### 2.3 Formulation of Gluten Free Noodles

**FIGURE 1:**
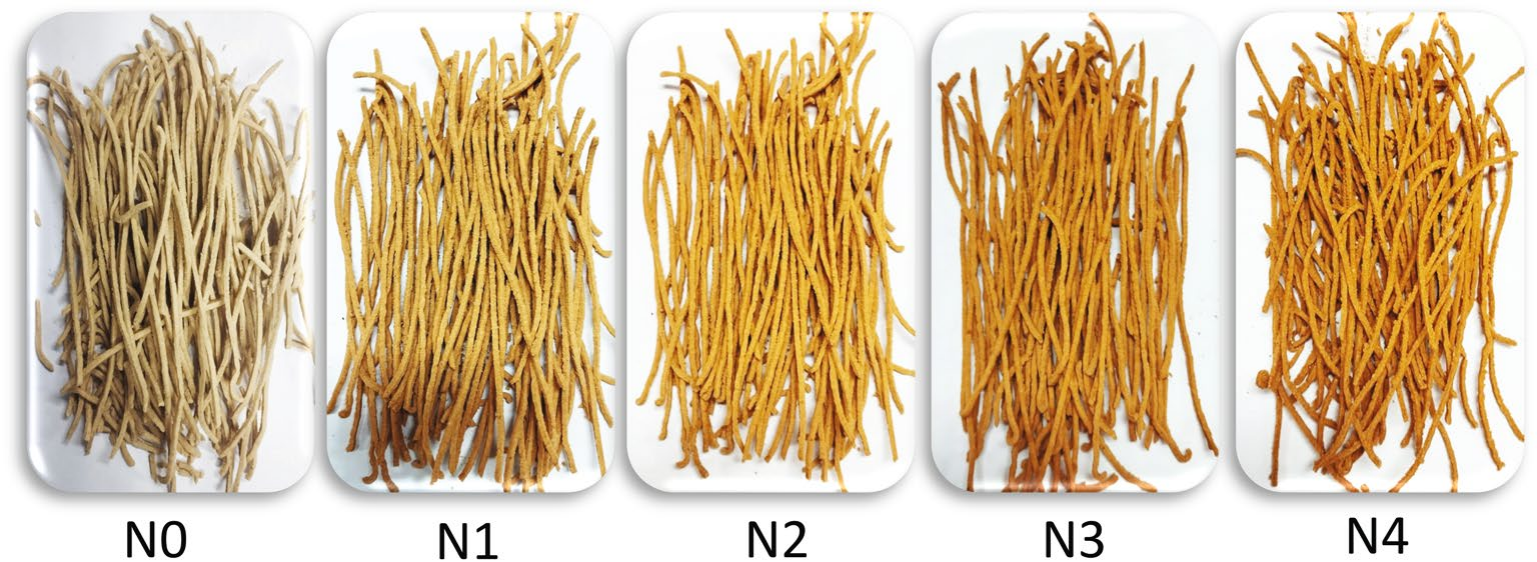
Illustration of formulated noodles. N0 = 100% FMF, N1 = 85% FMF + 5% TP + 10% DSF, N2 = 80% FMF + 10% TP + 10% DSF, N3 = 75% FMF + 15% TP + 10% DSF, N4 = 70% FMF + 20% TP + 10% DSF.

Noodles preparation was done according to the method described by Sarker et al. (2025) with slight modifications. The experimental noodles (Figure 1) were prepared by substituting FMF with varying proportions of TP: 5% (N1), 10% (N2), 15% (N3), and 20% (N4), while maintaining the DSF proportion at 10%. The control noodles were composed of 100% FMF (N0). The amount of cornstarch (2.0 g), CMC (2.0 g), xanthan gum (0.5 g), and salt (1.5 g) remained consistent for every 100 g of flour throughout all noodle variations. CMC and xanthan gum were incorporated following the study by (Culetu et al. 2021). All ingredients were homogenously mixed with warm water. Subsequently, the noodle dough underwent a kneading process for 10 min and was then allowed to rest for a duration of 30 min. The dough was uniformly processed with a manual noodle‐making apparatus (Changzhou Shule Kitchen Utensils Co. Ltd., China; LFGB certified, 150 mm, Stainless Steel 430) to produce the noodles sheet. This process facilitates obtaining the specified 2.0 mm of thickness and 2.5 mm of width of the noodle stick. Then, noodle sticks were obtained through cutting along the flattened surface of the sheet and subsequently oven dried at 60°C for about 6 h until a final moisture content of approximately 8%–10% was achieved. Hermetically sealed polyethylene bags were used to preserve dehydrated noodles in a cold, arid location until utilization.

### 2.4 Nutrient Composition Analysis

The nutritional analysis of the noodle samples was conducted using standard AOAC methods (2005). Specifically, moisture content was measured using method 925.10, protein using method 984.13A with a conversion factor of 6.25, fat by method 923.85, ash by method 923.03, crude fiber by method 920.86, and total dietary fiber using method 991.43 with the Megazyme Kit (K‐TDFR‐200A). All proximate analyses were performed in triplicate (*n* = 3). Carbohydrate content was calculated by deducting the combined percentages of moisture, protein, fat, and ash from 100. The energy content was determined using the following formula:

(1) $$\begin{array}{cc} \text{Total energy}\;\left(\frac{\text{kcal}}{100g}\right)= & [\left(\text{carbohydrate}+\text{protein}\right)\times 4\\ & +\left(\mathrm{fat}\times 9\right)+\left(\text{dietary fiber}\times 2\right)] \end{array}$$

### 2.5 Minerals Analysis

The mineral content (calcium, magnesium, sodium, zinc, manganese, copper and iron) in the noodles was determined using an atomic absorption spectrophotometer (AAS; ICS‐3000, Thermo, USA). Samples were first subjected to dry ashing in a muffle furnace at 650°C until a white ash was obtained and then dissolved using 3 mL of concentrated HCl (wet acid digestion) to prepare the solution for mineral quantification in accordance with AOAC (2005) methodology.

### 2.6 Extraction for Determination of Antioxidants Properties

For determination of antioxidant properties of noodle samples, extraction was done following the procedure outlined by Farzana et al. (2024). A mixture was formed by combining noodle powder (2 g) and methanol (20 mL), giving a solvent‐to‐sample ratio of 10:1 (v/w). Then, the mixture was agitated in a shaking incubator for 8 h (25°C) and subsequent centrifugation was done at 3360 *g* (relative centrifugal force) for 10 min. After collecting the supernatant, the volume was adjusted to 30 mL with methanol. All extractions were performed in triplicate (*n* = 3) to ensure reproducibility. For subsequent analyses, samples were stored at 4°C. Five different analyses, such as total phenolic content, total antioxidant content, total flavonoid content, ferric reducing antioxidant power (FRAP) assay, and DPPH free radical scavenging activity, were conducted to evaluate the antioxidant properties of noodles.

#### 2.6.1 Total Phenolic Content (TPC)

The Folin–Ciocalteu technique was employed to determine the TPC, with minor modifications (Sarker et al. 2025). In this procedure, 1 mL of 7.5% sodium carbonate solution, 4.5 mL of distilled water, and 1 mL of FC reagent were added to 0.5 mL of the noodle extracts. Subsequently, the mixture was allowed to rest for 30 min in a dark place at 25°C. The absorbance was measured at 765 nm wavelength by using a UV spectrophotometer. A standard GA curve was applied to determine the TPC, and the results were reported as mg GAE/100 g of sample.

#### 2.6.2 Total Flavonoids Content (TFC)

Total flavonoid content (TFC) was determined employing the technique detailed by Abedin et al. (2025) with minor adjustments. Firstly, 0.1 mL of 10% aluminum nitrate, 0.1 mL of 1 M potassium acetate and 4.5 mL of deionized water were added to sample extract (0.5 mL). The mixture was vortexed thoroughly and incubated at 25°C for 30 min., the absorbance was measured at 430 nm wavelength by using an UV spectrophotometer. The results were reported as mg QE/100 g of the sample.

#### 2.6.3 Total Antioxidants Capacity (TAC)

The TAC was measured following the methodology of Farzana et al. (2024) with slight adjustments. Initially, 0.5 mL of extracts was introduced into Falcon tube containing 5 mL of reagent solution consisting of 28 mM sodium phosphate, 0.6 M sulfuric acid and 4 mM ammonium molybdate. Subsequently, the tubes were incubated at 95°C for 90 min to promote the reaction. The absorbance was recorded at 695 nm with an UV spectrophotometer against a blank following the cooling of the sample. The standard AA calibration curve was used to calculate TAC. Total antioxidant capacity expressed in ascorbic acid equivalents (mg AAE/100 g).

#### 2.6.4 DPPH Radical Scavenging Activity

The DPPH radical‐scavenging activity was assessed following the technique outlined by Sarker et al. (2025) with minor changes. Initially, a solution of 1 mL of sample extract and 2 mL of 0.1 mM DPPH was taken in a Falcon tube. Subsequent incubation in darkness was done for 30 min. Two milliliters of DPPH and 1 mL of methanol were mixed to prepare a blank. Methanol was used as a blank. Then, the absorbance was measured at 517 nm by using a UV spectrophotometer. The below formula was employed to determine radical‐scavenging activity.

(2) $$\text{DPPH radical scavenging activity}\;\left(\%\right)=\frac{A_{0}-A_{s}}{A_{0}}\times 100$$

where *A*_0_ = absorbance of control and *A*_s_ = absorbance of sample.

#### 2.6.5 Ferric Reducing Antioxidant Power (FRAP)

The method outlined by Oyaizu (1986) was employed to perform the FRAP assay. For determining FRAP activity of sample extracts, 2.5 mL of phosphate buffer solution (0.2 M, pH 6.6) and 2.5 mL of potassium ferricyanide (1%, w/v) were added to 2.5 mL of noodle extracts. Subsequent incubation was performed at 50°C for 20 min. Following completion of the incubation time, 2.5 mL of a 10% TCA solution was introduced to each tube. The tubes were subsequently centrifuged at 3000 rpm for a duration of 10 min. The supernatant attained after centrifugation was subsequently amalgamated with deionized water (2.5 mL) in separate test tubes. 0.5 mL of FeCl_3_ solution (0.1% w/v) was included into the mixture, and the absorbance was recorded at 700 nm wavelength using a UV spectrophotometer. The FRAP values (mg AAE/100 g) were calculated using an ascorbic acid standard curve.

### 2.7 Color Test

The color test of the noodle samples was performed by A Minolta CR‐400 colorimeter according to the method described by Kasim and Kasim (2015) with slight adjustments. The color characteristics were determined using the CIELab system, where L* indicates lightness (ranging from 0 to 100), a* represents the red‐green axis [−a* (green) and +a* (red)], and b* corresponds to the blue‐yellow axis [−b* (blue) and +b* (yellow)]. Prior to measurement, the instrument was calibrated using a white standard plate (L* = 94.10; a* = −0.10; b* = 3.25). The results were expressed using the CIELab color space system, as established by the International Commission on Illumination. Each sample was measured five times to ensure accuracy. The obtained L*, a*, and b* values were further used to calculate browning index (BI), total color difference (Δ*E*), chroma, hue angle (*H*°), and whiteness index (WI), which were analyzed to determine color differences compared to the control sample.

(3) $$\mathrm{BI}=\frac{100\times \left(X-0.31\right)}{0.17}$$

where

(4) $$X=\frac{a^{*}+1.75\times L^{*}}{5.645\times L^{*}+a^{*}-3.012\times b^{*}}$$

(5) $$△E=\sqrt{{\left(L^{*}-L_{0}\right)}^{2}+{\left(a^{*}-a_{0}\right)}^{2}+{\left(b^{*}-b_{0}\right)}^{2}}$$

(6) $$\text{Chroma}=\sqrt{a^{*2}+b^{*2}}$$

(7) $$\mathrm{Hue}\;\text{angle}\;\left(H\right)=\tan^{-1}\left(a^{*}÷b^{*}\right)$$

(8) $$\mathrm{WI}=100-\sqrt{\;{\left(100-L^{*}\right)}^{2}+a^{*2}+b^{*2}}$$

where L*, a* and b* values are the measured values of the noodles incorporated with TP and DSF, and $L_{0}$, $a_{0}$ and $b_{0}$ are the measured values of the control noodles.

### 2.8 Texture Profile Analysis (TPA)

The texture profile analysis of noodles was conducted by implementing the methodology of (Koh et al. 2022) with a few adjustments. Noodles (30 g) were boiled (300 mL of water) for 7 min using an induction stove to evaluate their texture properties. Subsequent to cooking, boiled noodles were cooled for 30 s. A CTX texture analyzer (AMETEK Brookfield, USA) was utilized to analyze the texture profile. The test settings included a trigger force of 20 g, a pre‐test speed of 1 mm/s, a test speed of 1 mm/s, and a deformation of 1.5 mm. The characteristics calculated were hardness, cohesiveness, springiness index, adhesiveness, chewiness index, and gumminess. Measurements for each treatment were undergone 3 times.

### 2.9 Cooking Properties

#### 2.9.1 Water Absorption Capacity (WAC)

Ten grams of noodles were boiled in 150 mL of water. After a chilling period of 5 min in cold water, the remaining water was drained. Following formula was employed to calculate WAC (Farzana et al. 2024). WAC of noodles was performed in triplicate (*n* = 3) to ensure reproducibility.

(9) $$\mathrm{WAC}\;\left(g/100g\right)=\frac{W_{2}-W_{1}}{W}\times 100$$

Here, $W_{2}$ = weight of the cooked noodles (g); $W_{1}$ = weight of the raw noodles (g); *W* = weight of the raw noodles (g).

#### 2.9.2 Cooking Yield (CY)

The cooking yield of noodles was assessed by implementing the procedure outlined by Koh et al. (2022). Approximately 10 g of instant noodles were measured and boiled in 150 mL of distilled water at 100°C for 10 min while continuously stirred magnetically in a covered beaker. Subsequently, the cooked noodles were extracted from the boiling water, washed with cold water, drained, and allowed to cool for 15 min at room temperature (25°C) before weighing. CY of noodles was performed in triplicate (*n* = 3) to ensure reproducibility. Noodle's cooking yield was determined using the equation below:

(10) $$\text{Cooking yield}\;\left(g/100g\right)=\frac{W_{c}}{W}\times 100$$

Here, $W_{c}$ = weight of the cooked noodles (g); *W* = weight of the raw noodles (g).

#### 2.9.3 Cooking Loss (CL)

The cooking loss of noodles was evaluated using the technique applied in Koh et al. (2022). The water spent for cooking the noodles was transferred to a 250 mL volumetric flask and diluted to the calibration mark with distilled water. Following thorough mixing of the solution, a 10 mL aliquot was transferred into an aluminum dish and subjected to drying in an oven at 105°C until a stable weight was achieved. CL of noodles was performed in triplicate (*n* = 3) to ensure reproducibility. The cooking loss of the noodles was determined using the equation below:

(11) $$\text{Cooking loss}\;\left(g/100g\right)=\frac{W_{d}}{W}\times 100$$

Here, $W_{d}$ = weight of dried residue in cooking water (g); *W* = weight of raw noodles (g).

### 2.10 pH Measurement

pH level of the noodles was determined following the procedure outlined by (Koh et al. 2022). Noodles (2 g) and distilled water (10 mL) were thoroughly homogenized. The pH levels of the homogenized noodle samples were subsequently measured using a digital pH meter (HI2211 pH/ORP Meter, HANNA Instruments, Romania) at 25°C.

### 2.11 Sensory Attributes Analysis

Sensory properties of prepared noodles were evaluated following the method outlined by (Sarker et al. 2025) with minor adjustments. Initially, the cooked noodle samples were assigned random numbers and presented to the sensory panel in a randomized order within 5 min after cooking. Twenty trained panelists including 12 women and 8 men selected from the designated panel at the Institute of Food Science and Technology (IFST) under the Bangladesh Council of Scientific and Industrial Research (BCSIR) in Dhaka, Bangladesh, performed the sensory evaluation of prepared noodles. Each participant was positioned in their assigned sensory booth, where the assessment occurred. The panel leader assigned codes to the samples for identifying purposes. Each participant received a sensory assessment form that included color, texture, flavor, taste, mouthfeel, and overall approval. Additionally, the panelists were enquired about the overall acceptability of the noodles utilizing the same assessment form; however, in separate parts. The samples' quality was assessed on a 9‐point hedonic scale: 9 (like highly); 8 (like very much); 7 (like moderately); 6 (like slightly); 5 (neither like nor dislike); 4 (dislike slightly); 3 (dislike moderately); 2 (dislike very much); 1 (dislike highly). All participants submitted their written informed consent using a standard form throughout the assessment procedure. The head of the panel developed the report outlining the mean ± SD of the sensory assessment.

### 2.12 Statistical Analysis

The data was reported as the mean ± standard deviation (SD). A one‐way analysis of variance (ANOVA) was conducted using SPSS (version 22.0, IBM, Chicago, IL, USA) for determining statistically significant differences in means (*p* < 0.05). Bonferroni post hoc test was applied to identify pairwise differences between groups. Origin 2024 software was used for graphical representation.

## 3 Results and Discussions

### 3.1 Nutrient Composition of Noodles

The results of the nutrient composition of all the noodle samples are outlined in Table 1. The moisture content of TP and DSF enriched noodles ranged from 4.38 ± 0.04 to 4.70 ± 0.02 g/100 g whereas N0 had 4.18 ± 0.04 g/100 g. This increase may be attributed to the higher water‐binding ability of TP and DSF, which enhances moisture retention in the product (Aleem Zaker et al. 2012; El Makhzangy et al. 2024). N1, N2, N3, and N4 exhibited substantially (*p* < 0.05) greater content of ash (2.93 ± 0.25 to 5.04 ± 0.03 g/100 g) in comparison to N0 (2.21 ± 0.08 g/100 g). Each increase in TP led to a significant (*p* < 0.05) rise in ash content, which might be ascribed to higher ash content in TP (3.53 g/100 g) compared to FMF (1.39 g/100 g) (Abedin et al. 2022). This finding aligns with another study (Nakov et al. 2022) that reported a substantial rise in ash content in crackers with 4%, 6%, 8%, and 10% TP enrichment. In the present study, adding DSF also contributed to the ash content, as soybeans contain plenty of minerals (Etiosa et al. 2017). The TP and DSF enriched noodles had crude fiber levels between 2.78 ± 0.07 and 3.83 ± 0.09 g/100 g, which is substantially (*p* < 0.05) higher than N0 (2.09 ± 0.13 g/100 g). Though FMF itself contains a good amount of crude fiber, the incorporation of tomato powder in the experimental formulations, which contain a comparatively higher amount of crude fiber (6.37 g/100 g) (Ladi et al. 2017) than that of FMF (2.21 g/100 g) (Abedin et al. 2022), further enhanced the fiber content in noodles. The incorporation of 1% moringa leaf powder and 2% tomato powder was reported to elevate the crude fiber content of soybean chips (Harika et al. 2024), while the addition of 10%, 20%, and 30% DSF was reported to increase the total crude fiber content (0.5 to 0.8 g/100 g) of biscuits (Aleem Zaker et al. 2012) by replacing conventional flour. However, crude fiber only represents part of the total dietary fiber and may not fully reflect digestive health benefits. Therefore, total dietary fiber content in noodles has been measured and discussed in Section 3.2. The protein content in enriched noodles increased from 16.87 ± 0.07 to 17.21 ± 0.04 g/100 g, indicating that all the experimental noodles had significantly (*p* < 0.05) more protein than the control (12.34 ± 0.10 g/100 g). This rise in protein levels in all the experimental recipes compared to N0 is due to the incorporation of DSF, which has high protein (48.9%) content (Mashayekh et al. 2008). A significantly decreased (*p* < 0.05) fat content (3.33 ± 0.02 to 2.85 g/100 ± 0.03 g) was observed in the TP and DSF enriched noodles compared to the control (3.66 ± 0.03 g/100 g). The range in the amount of carbohydrate was 76.87 ± 0.36 to 74.90 ± 0.01 g/100 g (N1 to N4), which is significantly lower compared to N0 (81.80 ± 0.23 g/100 g). The energy values varied from 404.93 ± 0.98 to 394.07 ± 0.25 kcal/100 g (N1 to N4), which is notably (*p* < 0.05) lower compared to N0 (409.48 ± 0.25 kcal/100 g). The enhanced levels of protein, fiber, and ash in the noodles present a superior choice for those seeking plant‐derived gluten‐free options. Additionally, increased protein in noodles might enhance their structural integrity through the formation of a stronger gel matrix during cooking, while increased fiber might contribute to greater water‐binding capacity of noodles, which may increase cooking yield.

**TABLE 1:** Nutrient composition of noodles (dry basis).

Nutrient (per 100 g)	N0	N1	N2	N3	N4
Moisture (g)	4.18 ± 0.04^d^	4.38 ± 0.04^c^	4.51 ± 0.06^b^	4.63 ± 0.02^a^	4.70 ± 0.02^a^
Ash (g)	2.21 ± 0.08^e^	2.93 ± 0.25^d^	3.68 ± 0.04^c^	4.39 ± 0.03^b^	5.04 ± 0.03^a^
Protein (g)	12.34 ± 0.10^d^	16.87 ± 0.07^c^	17.00 ± 0.03^bc^	17.10 ± 0.04^ab^	17.21 ± 0.04^a^
Fat (g)	3.66 ± 0.03^a^	3.33 ± 0.02^b^	3.28 ± 0.02^b^	3.06 ± 0.04^c^	2.85 ± 0.03^d^
Crude fiber (g)	2.09 ± 0.13^e^	2.78 ± 0.07^d^	3.12 ± 0.10^c^	3.43 ± 0.09^b^	3.83 ± 0.09^a^
Dietary fiber (g)	16.43 ± 0.03^e^	17.94 ± 0.17^d^	20.02 ± 0.02^c^	22.76 ± 0.04^b^	24.50 ± 0.04^a^
Carbohydrate (g)	81.80 ± 0.23^a^	76.87 ± 0.36^b^	76.03 ± 0.05^c^	75.46 ± 0.13^cd^	74.90 ± 0.01^d^
Energy (Kcal)	409.48 ± 0.24^a^	404.93 ± 0.98^b^	401.69 ± 0.04^c^	397.75 ± 0.02^d^	394.07 ± 0.25^e^
Na (mg)	441.12 ± 2.82^d^	499.32 ± 8.93^c^	517.08 ± 5.49^c^	540.17 ± 5.68^b^	561.06 ± 8.13^a^
K (mg)	423.86 ± 5.09^e^	707.55 ± 7.07^d^	852.17 ± 4.32^c^	983.14 ± 4.76^b^	1107.47 ± 8.80^a^
Ca (mg)	47.24 ± 3.92^d^	77.67 ± 1.11^c^	83.42 ± 3.24^bc^	89.58 ± 3.52^ab^	95.07 ± 4.31^a^
Mg (mg)	46.54 ± 3.86^d^	77.38 ± 5.12^c^	85.75 ± 1.31^bc^	89.80 ± 3.18^ab^	99.23 ± 1.53^a^
Fe (mg)	4.70 ± 0.25^a^	4.83 ± 0.08^a^	4.79 ± 0.11^a^	4.77 ± 0.07^a^	4.70 ± 0.09^a^
Cu (mg)	0.54 ± 0.07^c^	0.77 ± 0.06^b^	0.84 ± 0.02^b^	0.89 ± 0.02^ab^	1.02 ± 0.04^a^
Zn (mg)	2.48 ± 0.04^d^	2.56 ± 0.02^bc^	2.61 ± 0.02^bc^	2.64 ± 0.02^ab^	2.71 ± 0.03^a^
Mn (mg)	0.73 ± 0.03^d^	0.95 ± 0.03^c^	1.01 ± 0.02^c^	1.11 ± 0.02^b^	1.25 ± 0.05^a^

*Note:* N0 = 100% FMF, N1 = 85% FMF + 5% TP + 10% DSF, N2 = 80% FMF + 10% TP + 10% DSF, N3 = 75% FMF + 15% TP + 10% DSF, N4 = 70% FMF + 20% TP + 10% DSF. Values are means of triplicates ± standard deviation. A one‐way ANOVA was conducted, followed by Bonferroni test for statistical analysis. The mean values denoted by distinct lowercase letters in each row are significantly different (*p* < 0.05).

Abbreviations: DSF = defatted soya flour, FMF = foxtail millet flour, TP = tomato powder.

### 3.2 Total Dietary Fiber

Dietary fiber constitutes the indigestible component of plant‐derived meals, including fruits, vegetables, whole grains, legumes, and nuts, and aids in reducing blood cholesterol and glucose levels and facilitating regular bowel motions (He et al. 2022). They play significant role in noodles matrix and texture by modifying water absorption, dough viscosity and the structural arrangement of starch and protein components (Pakhare et al. 2018). Table 1 presents the dietary fiber content of all the noodles in the study. In comparison to N0 (16.43 ± 0.03 g/100 g), N1–N4 exhibited a substantial (*p* < 0.05) elevation in dietary fiber content (17.94 ± 0.17 to 24.50 ± 0.04 g/100 g). Each increase in TP led to a notable (*p* < 0.05) rise in dietary fiber, potentially attributable to the greater dietary fiber present in whole tomatoes, which also includes tomato peel and seed (Niu et al. 2018; Li et al. 2022). The additional fiber might contribute to enhancing water retention, leading to a softer matrix and reduced hardness of cooked noodles (discussed in Section 3.6 texture profile analysis of noodles). The incorporation of 4%–10% TP was reported to elevate total dietary fiber content (4.08 to 7.80 g/100 g) of cream crackers (Nakov et al. 2022). Some fiber works as prebiotics, supporting beneficial gut flora and promoting immunological function. For instance, prebiotic properties of tomato flour were reported in a study by (Coelho et al. 2023) through the modulation of gut microbiota. Consistently eating fiber‐dense meals is crucial for disease prevention and general health (He et al. 2022; Snauwaert et al. 2023).

### 3.3 Mineral Composition of Noodles

Minerals are essential nutrients necessary for organisms, including humans, to perform vital processes for life and health. Essential nutrients are termed so because they cannot be produced by the body and must be obtained from dietary sources or, in exceptional instances, supplementation (Awuchi et al. 2020). In the present study, Table 1 delineates the mineral composition of all the formulated noodles, indicating that the content of calcium, magnesium, sodium, and potassium in N1–N4 was considerably elevated (*p* < 0.05) compared to N0. Calcium level in enriched noodles (77.67 ± 1.11 to 95.07 ± 4.31 mg/100 g) was significantly (*p* < 0.05) higher compared to N0 (47.24 ± 3.92 mg/100 g). Magnesium level ranged from 77.38 ± 5.12 to 99.23 ± 1.53 mg/100 g (N1–N4), whereas the control noodle had significantly (*p* < 0.05) lower value (46.54 ± 3.86 mg/100 g). Sodium level varied from 499.32 ± 8.93 to 561.06 ± 8.13 mg/100 g (N1–N4), indicating notably (*p* < 0.05) higher values compared to N0 (707.55 ± 7.07 mg/100 g). Similarly, a significant (p < 0.05) increase in potassium level was observed, varying from 441.12 ± 2.82 to 423.86 ± 5.09 mg/100 g (N1–N4) compared to N0 (1107.47 ± 8.80 mg/100 g). The zinc levels varied from 2.56 ± 0.02 to 2.71 ± 0.03 mg/100 g whereas N0 had 2.48 ± 0.04 only. Copper levels in the formulated noodles ranged between 0.77 ± 0.06 mg/100 g and 1.02 ± 0.04 mg/100 g, which is notably higher (*p* < 0.05) than that of N0 (0.54 ± 0.07). The manganese levels ranged from 0.95 ± 0.03 to 1.25 ± 0.05 mg/100 g, indicating significantly (*p* < 0.05) higher level compared to the N0 (0.73 ± 0.03). However, the Fe content didn't differ significantly among all the noodles. This significant rise in mineral content of experimental noodles compared to control noodles might be attributed to good mineral content in tomato powder (Srivastava and Kulshreshtha 2013) and soy flour (Etiosa et al. 2017). Although mineral bioavailability was not evaluated in this study, the formulation clearly improves the mineral content of the noodles. The absorption of these minerals and thus their full health‐promoting potential remains to be determined in future investigations.

### 3.4 Antioxidants Properties of Noodles

Antioxidants comprise phenolics, flavonoids, carotenoids, Vitamin C, Vitamin E, etc. which are essential components, safeguarding the body against oxidative stress induced by free radicals, which can harm cells, proteins, and DNA (Pisoschi and Pop 2015). A diet abundant in antioxidants may aid in the prevention of heart disease, cancer, cognitive decline, and strengthening of the immune system (Zhang et al. 2015; Griffiths et al. 2016). Gluten‐free products frequently lack antioxidants, driving an increasing interest in formulating antioxidant‐rich gluten‐free diets by integrating antioxidant‐rich components (Torres et al. 2017). Therefore, incorporation of tomato powder, rich in lycopene and other carotenoids, and soybean flour, abundant in phenolics and isoflavones, may mechanistically enhance the antioxidant capacity of the gluten‐free diet (Yang et al. 2023).

#### 3.4.1 Total Phenolic Content (TPC)

**FIGURE 2:**
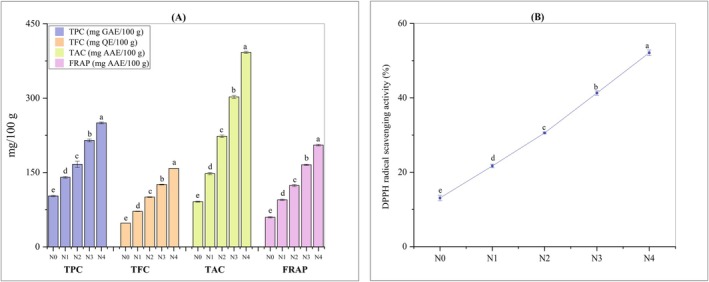
Antioxidant properties of noodles: (A) TPC, TFC, TAC, and FRAP (B) DPPH radical scavenging activity. N0 = 100% FMF, N1 = 85% FMF + 5% TP + 10% DSF, N2 = 80% FMF + 10% TP + 10% DSF, N3 = 75% FMF + 15% TP + 10% DSF, N4 = 70% FMF + 20% TP + 10% DSF, AAE = ascorbic acid equivalent, DPPH = 2,2‐diphenyl‐1‐picryl‐hydrazyl, FRAP = ferric reducing antioxidant power, GAE = gallic acid equivalent, QE = quercetin equivalent, TAC = total antioxidant content, TFC = total flavonoid content, TPC = total phenolic content. Values are means of triplicates ± standard deviation. The statistical analysis was performed using a one‐way ANOVA. The mean values with different lowercase letters in each column and line graph are significantly (*p* < 0.05) different.

Phenolic compounds e.g., flavonoids, phenolic acids, lignans, tannins, coumarins, etc. represent a category of metabolites that originate from the secondary pathways within plants such as cereals, vegetables, fruits, leaves, and roots (Zagoskina et al. 2023). Phenolic constituents demonstrate several biological actions, including anti‐inflammatory, antibacterial, anticancer, and anti‐aging (Rahman et al. 2021). In the present study, Figure 2A represents TPC of noodle samples. The incorporation of varying percentages of TP and DSF significantly (*p* < 0.05) improved the TPC of noodles (140.32 ± 1.86 mg GAE/100 g, 166.56 ± 6.26 mg GAE/100 g, 214.77 ± 2.88 mg GAE/100 g, and 250.05 ± 1.88 mg GAE/100 g for N1, N2, N3, and N4, respectively) when compared to the control noodle (102.69 ± 1.27 mg GAE/100 g). This is likely attributed to the presence of polyphenols and phenolic acid in tomatoes (Wang et al. 2023). DSF also contributed to the TPC of formulated noodles, as soybeans are a beneficial source of phenolic compounds (Krol‐Grzymała and Amarowicz 2020). These findings are comparable with other studies, such as integration of 10% and 20% of tomato pomace flour into durum wheat dough, which led to a significant (*p* < 0.05) increase in TPC of functional bread (Brighina et al. 2024). Another study (Estivi et al. 2024) reported a substantial (*p* < 0.05) increase in phenolics, carotenoids, and antioxidant activity in gluten‐free pastas after enrichment with tomato waste (10% and 15%), compared to the control formulation consisting of fava bean (33.3%) and rice (66.7%) flour.

#### 3.4.2 Total Flavonoid Content (TFC)

Flavonoids, a category of natural compounds characterized by diverse phenolic structures and recognized for their health benefits, are present in fruits, vegetables, cereals, and other sources. The TFC of the various noodle types is presented in Figure 2A, which demonstrates that TP and DSF integration significantly (*p* < 0.05) elevated the TFC in TP and DSF enriched noodles, ranging from 71.64 ± 0.49 to 158.28 ± 0.08 mg QE/100 g with the highest value found in N4, compared to the control (48.02 ± 0.23 mg QE/100 g). This rise in flavonoids, particularly those derived from tomato, may also contribute to the reddish coloration of the enriched noodles and influence sensory perception by enhancing attributes such as flavor intensity and overall visual appeal (Kurina et al. 2021; Jeyaprakash et al. 2020). A comparable pattern was noted by another study, suggesting a dose‐dependent enhancement in flavonoid levels with 2%, 4%, 6%, and 8% of tomato powder in cookies (Hwang and Cheung 2024). Yagci et al. (2022) demonstrated that enhancing the concentration of tomato pomace powder in extruded snacks markedly elevated the quantities of certain free phenolics, such as gallic acid, chlorogenic acid, rutin, 2,5‐dihydroxybenzoic acid, protocatechuic acid, and quercetin. DSF also contributed to the TFC of formulated noodles, as soybeans are a beneficial source of flavonoids, particularly rich in isoflavones, which have potential for cancer prevention (Malencic et al. 2012). The pharmacological evidence documented in the literature demonstrates that flavonoids exhibit a range of activities, including anti‐cancer, anti‐microbial, anti‐oxidant, anti‐inflammatory, anti‐fungal, anti‐ulcer, and anti‐edematogenic properties (Ekalu and Habila 2020). Therefore, formulated noodles enriched with flavonoids might be considered as potential, supporting long‐term health enhancement.

#### 3.4.3 Total Antioxidant Capacity (TAC)

Natural antioxidants derived from plant sources mainly comprise polyphenols (phenolic acids, flavonoids, lignans, anthocyanins, and stilbenes), carotenoids (xanthophylls and carotenes), and vitamins (vitamins E and C). These compounds demonstrate a diverse array of biological effects, including anti‐inflammatory, antibacterial, anticancer, anti‐aging, and antiviral properties (Xu et al. 2017). Therefore, efforts have been undertaken to optimize the production of foods enhanced with antioxidants and antioxidant‐like compounds while preserving their organoleptic and structural attributes. In the present study, the impact of TP and DSF incorporation on the TAC of noodles was studied, and the results are presented in Figure 2A. The TAC of N1–N4 varied from 147.88 ± 2.29 to 392.32 ± 2.08 mg AAE/100 g, which is considerably greater (*p* < 0.05) than that of the N0 (91.12 ± 1.07 mg AAE/100 g). As the proportion of TP increased, a significant (*p* < 0.05) increasing trend was observed across the experimental noodles, demonstrating an enhancing effect of TP incorporation on TAC. This improvement can be attributed to bioactive components, such as β‐carotene, lycopene, flavonoids, phenolics present in tomatoes, all of which contribute to antioxidants activity (Tilahun et al. 2021). Refined flour partially replaced with 35% and 40% tomato pomace in bread and muffins resulted in a substantial (*p* < 0.05) enhancement of antioxidant activity (Mehta et al. 2018). A study by Bhat et al. reported that enrichment of cakes with tomato powder led to a significant improvement in antioxidant properties (*p* < 0.05) (Bhat et al. 2022). Formulated noodles' TAC was also contributed by DSF, as soybeans are a good source of antioxidants (Rizzo 2020).

#### 3.4.4 DPPH Radical Scavenging Activity

Antioxidants possess radical scavenging properties through termination of free‐radical chain reactions by scavenging reactive species such as peroxide radicals, thereby improving the quality and stability of food. Figure 2B illustrates the DPPH scavenging activity of noodle samples, emphasizing the positive effect on DPPH scavenging activity noted in the noodles enhanced with TP. The inclusion of varying percentages of TP and DSF significantly (*p* < 0.05) increased the DPPH scavenging activity of the noodles, measuring 21.7% ± 0.49%, 30.57% ± 0.22%, 41.30% ± 0.64%, and 52.12% ± 0.76% for N1, N2, N3, and N4, respectively, compared to N0 (13.09%). The results indicated that the control noodle had the least DPPH scavenging activity when compared to all other samples. Each increase in the percentage of TP exhibited a substantial (*p* < 0.05) enhancing impact on DPPH across the experimental samples. This improved scavenging capacity might be attributed to the synergistic effect of lycopene, carotenoids, and phenolic compounds abundant in tomato and isoflavones and phenolics from DSF, which can scavenge DPPH radicals by donating hydrogen atoms (Liu et al. 2008). A notable rise in antioxidant capacity (*p* < 0.05) was found in pastas (gluten‐free) that included 10% and 15% tomato waste, compared to the control recipe made of 33.3% fava bean flour and 66.7% rice flour (Estivi et al. 2024). A gluten‐free snack (ready‐to‐cook), composed of potato flour and finger millet, was supplemented with 10% tomato pomace powder, leading to a significant (*p* < 0.05) increase in DPPH and FRAP activity (Rehal et al. 2022).

#### 3.4.5 Ferric Reducing Antioxidant Power (FRAP)

The FRAP assay is a commonly used method for quantifying the antioxidant activity of reductive antioxidants in a sample (Benzie and Devaki 2018). The present study investigated the effect of TP and DSF inclusion on FRAP activity of noodles, with findings presented in Figure 2A. The inclusion of TP and DSF significantly enhanced the FRAP antioxidant activity of the noodles (*p* < 0.05), with values ranging from 94.96 ± 1.28 to 205.30 ± 1.78 mg AAE/100 g, compared to 59.73 ± 1.44 mg AAE/100 g in the control (N0). This finding aligns with another research where a rapid ready‐to‐cook gluten‐free snack, composed of finger millet and potato flour, was supplemented with 10% tomato pomace powder, leading to a significant (*p* < 0.05) increase in DPPH and FRAP activity (Rehal et al. 2022). The consistent improvement in both assays (FRAP and DPPH) might be due to dual antioxidant mechanisms by lycopene, carotenoids, and phenolic compounds from TP, acting both as electron donors (enhancing FRAP) and hydrogen atom donors (enhancing DPPH). A significant rising trend was noticed in the FRAP activity of crackers with the increase of tomato pomace powder incorporation (Nakov et al. 2022). A substantial increase was observed in FRAP activity in pastas (gluten‐free) enriched with tomato waste (10% and 15%), compared to the control formulation consisting of 33.3% fava bean flour and 66.7% rice flour (Estivi et al. 2024). A diet abundant in antioxidants may aid in the prevention of cancer, cognitive decline, heart disease, and strengthening of the immune system (Zhang et al. 2015; Griffiths et al. 2016).

### 3.5 Color Properties of Noodles

Color and appearance are essential quality factors influencing consumer acceptance of noodle products. It also corresponds with type and quality of raw materials, and product's processing conditions. Research demonstrates that the integration of locally sourced vegetable or fruit powder into food products, such as bread and noodles, has resulted in desirable color aligned with consumer preferences (Farzana et al. 2024; Abedin et al. 2025). The color profile of all the formulated noodles is outlined in Table 2. In general, all the enriched noodles exhibited significantly decreased (*p* < 0.05) lightness (L* = 62.49 ± 0.02 to 53.86 ± 0.12), increased redness (a* = 6.76 ± 0.03 to 11.23 ± 0.07) and yellowness values (b* = 31.71 ± 0.02 to 33.47 ± 0.60) in comparison to N0 (L* = 68.42 ± 0.01; a* = 2.62 ± 0.02; b* = 25.14 ± 0.06). Additionally, L* and a* values exhibited considerably (*p* < 0.05) rising pattern with the increase of TP across the experimental formulations. Incorporation of tomato pomace powder into bread, muffin and crackers led to darker, more red and yellowish color (Modzelewska‐Kapituła 2012; Mehta et al. 2018). The increasing pattern of redness and yellowness in the experimental noodles might be attributed to the carotenoids, anthocyanins, chlorophyll present in tomato powder (Kurina et al. 2021). Furthermore, significantly (*p* < 0.05) higher values in BI (76.36 ± 0.05 to 107.01 ± 2.47), Δ*E* (9.76 ± 0.02 to 18.86 ± 0.27) and chroma (32.42 ± 0.01 to 35.31 ± 0.52) were noticed in all the experimental noodles compared to N0 (BI = 47.5 ± 0.15; Δ*E* = 0.00; chroma = 25.28 ± 0.07). However, WI values exhibited significantly (*p* < 0.05) decreasing pattern in N1–N4 compared to N0. In case of hue angle, N2–N4 showed significantly (*p* < 0.05) lower values than N0. From a practical standpoint, the darker and more vibrant color did not negatively affect sensory outcomes. In fact, formulations with moderate TP levels, particularly N2 and N3 got higher scores in terms of color during sensory evaluation (described in Section 3.9) which is consistent with another study (Concha‐Meyer et al. 2019).

**TABLE 2:** Color properties of noodles.

Sample	N0	N1	N2	N3	N4
L*	68.42 ± 0.01^a^	62.49 ± 0.02^b^	59.19 ± 0.19^c^	55.54 ± 0.21^d^	53.86 ± 0.12^e^
a*	2.62 ± 0.02^e^	6.76 ± 0.03^d^	8.47 ± 0.04^c^	10.54 ± 0.15^b^	11.23 ± 0.07^a^
b*	25.14 ± 0.06^c^	31.71 ± 0.02^b^	32.07 ± 0.03^b^	32.97 ± 0.10^a^	33.47 ± 0.60^a^
Browning index (BI)	47.51 ± 0.15^e^	76.36 ± 0.05^d^	85.58 ± 0.27^c^	99.64 ± 0.98^b^	107.01 ± 2.47^a^
Total color difference (Δ*E*)	0.00	9.76 ± 0.02^d^	12.93 ± 0.11^c^	17.03 ± 0.25^b^	18.86 ± 0.27^a^
Chroma	25.28 ± 0.07^d^	32.42 ± 0.01^c^	33.17 ± 0.03^b^	34.62 ± 0.13^a^	35.31 ± 0.52^a^
Hue angle	1.47 ± 0.00^a^	1.36 ± 0.00^a^	1.31 ± 0.00^b^	1.26 ± 0.00^c^	1.25 ± 0.01^d^
WI	59.55 ± 0.04^a^	50.42 ± 0.02^b^	47.41 ± 0.13^c^	43.65 ± 0.23^d^	41.90 ± 0.39^e^

*Note:* N0 = 100% FMF, N1 = 85% FMF + 5% TP + 10% DSF, N2 = 80% FMF + 10% TP + 10% DSF, N3 = 75% FMF + 15% TP + 10% DSF, N4 = 70% FMF + 20% TP + 10% DSF. Values are means of triplicates ± standard deviation. A one‐way ANOVA was conducted, followed by Bonferroni test for statistical analysis. The mean values denoted by distinct lowercase letters in each row are significantly different (*p* < 0.05).

Abbreviations: DSF = defatted soya flour, FMF = foxtail millet flour, TP = tomato powder.

### 3.6 Texture Profile Analysis of Noodles

**FIGURE 3:**
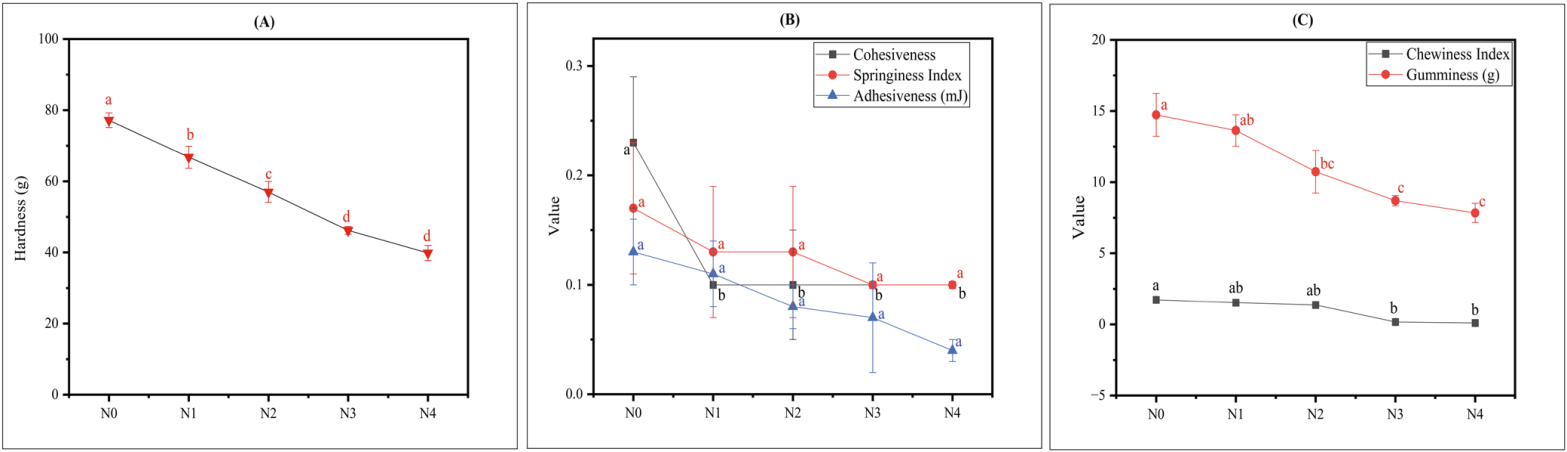
Texture profile analysis of noodles. (A) Hardness, (B) cohesiveness, springiness index, adhesiveness and (C) chewiness index, gumminess. N0 = 100% FMF, N1 = 85% FMF + 5% TP + 10% DSF, N2 = 80% FMF + 10% TP + 10% DSF, N3 = 75% FMF + 15% TP + 10% DSF, N4 = 70% FMF + 20% TP + 10% DSF. The statistical analysis was performed using a one‐way ANOVA. The mean values with different lowercase letters in each line graph are significantly (*p* < 0.05) different.

The current study examined the impact of TP and DSF incorporation on the texture profile of noodles, with results reported in Figure 3. The hardness of noodles enriched with TP and DSF varied from 66.77 ± 3.09 g to 39.83 ± 2.12 g, whereas the hardness of N0 was 77.17 ± 2.05 g, demonstrating a significant (*p* < 0.05) decline following the addition of TP and DSF. Incorporation of tomato powder increased the dietary fiber of noodles (Table 2), which may disrupt the growth of the starch gel network after heating. Inclusion of insoluble dietary fiber (IDF) weakens the hardness of noodles by disrupting the gluten starch network; the molecular interactions between IDF and starch during gelatinization involve hydrogen bonding with hydroxyl groups, influencing moisture migration and water absorption (Bhatt and Gupta 2023). Cohesiveness scores significantly (*p* < 0.05) fall in TP and DSF‐enriched noodles compared to N0. Springiness decreased slightly with no statistically significant (*p* < 0.05) difference compared to N0. The declining trend may result from the disruption of the starch network by increased fibers and proteins resulted from TP and DSF. Decreased starch concentration affects gel elasticity (Castanha et al. 2021). Adhesiveness declined in TP and DSF enriched noodles compared to N0. This decline might be attributable to a drier texture resulting from the water‐binding capability of tomato powder, which leads to less gelatinized starch on the surface. However, no statistically significant (*p* < 0.05) difference was noticed. Chewiness declined after the incorporation of TP and DSF into the noodles; however, only N3 and N4 exhibited significant differences (*p* < 0.05) when compared to N0. Chewiness is equal to gumminess multiplied by springiness, which aligns with observed patterns (Chandra and Shamasundar 2015). Noodles with reduced hardness and chewiness may facilitate chewing, possibly advantageous for specific demographics (e.g., children or the elderly). Additionally, the integration of TP and DSF resulted in a significant (*p* < 0.05) decrease in gumminess in noodles (N2–N4) compared to N0. These findings align with the sensory results (Section 3.9) which demonstrates that moderate incorporation of TP and DSF (up to 15% TP) softens the noodle matrix without adversely affecting sensory acceptance, whereas excessive levels (20% TP) may compromise textural quality and consumer acceptance.

### 3.7 Cooking Properties of Noodles

**FIGURE 4:**
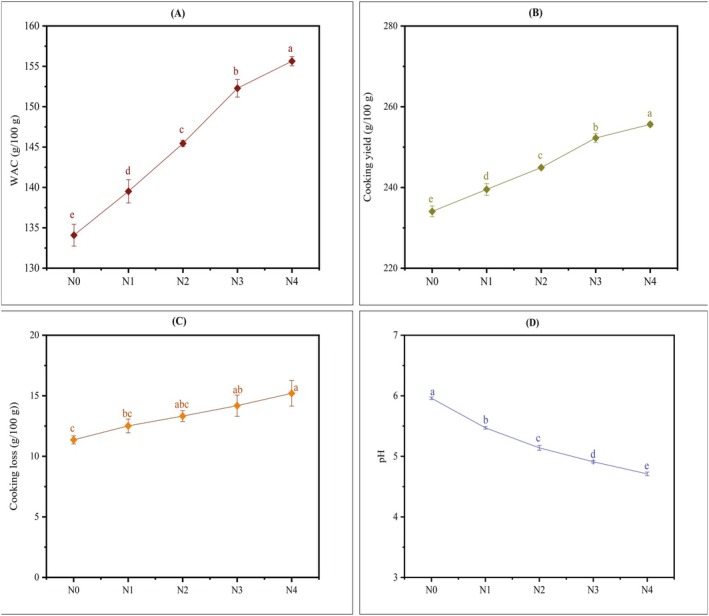
Cooking properties and pH of noodles: (A) WAC, (B) Cooking yield, (C) Cooking Loss, and (D) pH. N0 = 100% FMF, N1 = 85% FMF + 5% TP + 10% DSF, N2 = 80% FMF + 10% TP + 10% DSF, N3 = 75% FMF + 15% TP + 10% DSF, N4 = 70% FMF + 20% TP + 10% DSF, WAC = water absorption capacity. Values are means of triplicates ± standard deviation. The statistical analysis was performed using a one‐way ANOVA. The mean values with different lowercase letters in each line graph are significantly (*p* < 0.05) different.

Noodles' standard parameters utilized to assess cooking properties comprise water absorption capacity, cooking yield, and cooking loss. Cooking properties of noodle samples are depicted in Figure 4.

The data depicted in Figure 4A reveal a significant rise (*p* < 0.05) in water absorption capacity (WAC) with the incorporation of TP and DSF. The WAC of all experimental noodles ranged from 139.52 ± 1.45 to 155.64 ± 0.58 g/100 g, significantly (*p* < 0.05) greater than the N0 (134.08 ± 1.35 g/100 g). This increase in the WAC of noodles matrix led to a notable rise in the cooking yield (CY) of noodles which is depicted in Figure 4B. The CY of all experimental noodles varied between 239.52 ± 1.45 and 255.64 ± 0.58 g/100 g, substantially (*p* < 0.05) above N0 (234.08 ± 1.35 g/100 g), with N4 exhibiting the highest CY (255.64 g/100 g) among all samples prepared. Tomato powder is abundant in both soluble and insoluble fibers, including pectin and cellulose and dietary fiber possesses a significant capability to bind and retain water (Mpalanzi et al. 2023), hence enhancing the total water‐holding capacity of the noodle matrix. The water absorption of flour rose with the elevation of tomato pomace powder levels which was ascribed to the high concentrations of hydrocolloids in tomato pomace powder as the presence of hydrophilic groups inside the hydrocolloid structure facilitates increased water contact via hydrogen bonding (Majzoobi et al. 2011). In another study, incorporation of soy flour increased the WAC of soup powder because of the higher protein present in soy flour which can bind substantial amounts of water due to its capacity to establish hydrogen bonds between molecules and the polar groups on the polypeptide chain (Mohajan et al. 2018). The increase in the proportion of fiber‐rich flours (pumpkin flour) in noodles led to an increase in WAC and thereby CY, according to (Farzana et al. 2023). The swelling of noodles could facilitate delayed digestion and increased satiety, indicating that these noodles are a functional food. Additionally, noodles that offer a greater cooking yield might lead to enhanced nutrient retention (Showell et al. 2012) throughout the cooking process. According to Figure 4C, a slight variation was observed in the CL of TP and DSF‐enriched noodles and control noodles. The CL in experimental noodles varied from 12.51 ± 0.57 to 15.20 ± 1.06 g/100 g whereas the CL in N0 was 11.36 ± 0.34 g/100 g. The CL in N3 and N4 exhibited considerably (*p* < 0.05) elevated values compared to N0. However, there was no significant (*p* < 0.05) difference observed in the case of N1 and N2 when compared to N0. This increase in cooking loss is likely due to the soluble fibers and proteins from tomato powder and defatted soybean flour, which can disrupt the starch‐protein matrix and enhance the solubility of certain components during cooking. Such an increase in CL might increase nutrients loss by releasing advantageous bioactive components into the cooking medium, leading to lower nutrient retention if discarded. Incorporating fiber‐rich flours for example, pumpkin flour into noodles led to a significant (*p* < 0.05) increase in CL across samples, according to another study (Farzana et al. 2023).

### 3.8 pH of Noodles

pH is an important physicochemical parameter that significantly impacts the safety, quality, stability, and sensory attributes of food products (Andres‐Bello et al. 2013). The majority of bacteria thrive in neutral to mildly acidic environments (pH 6.0–7.5), but acidic conditions can prevent microbial growth, thereby prolonging shelf life (Alegbeleye et al. 2022). In the present study, pH values are reported in Figure 4D. The pH of all experimental noodles varied from 5.47 ± 0.02 to 4.73 ± 0.03, considerably (*p* < 0.05) lower than the pH of the control (5.96 ± 0.02). Each increase in the amount of TP resulted in a substantial (*p* < 0.05) decrease in the pH of noodles, revealing that the acidity of noodles increased with the incorporation of TP into noodles. Other research supports this finding where the pH of frankfurters decreased with increasing the concentrations of tomato powder and the acidic characteristics of the tomato powder (pH: 4.48–5.02) were reported as a potential explanation (Eyiler and Oztan 2011). This acidic pH might keep impacts on microbial inhibition, extended shelf life, flavor, color, texture, etc. (Andres‐Bello et al. 2013). Additionally, overall product stability depends on the combined effect of pH and other factors such as water activity, packaging, and storage conditions (Li et al. 2017).

### 3.9 Sensory Attributes of Noodles

**FIGURE 5:**
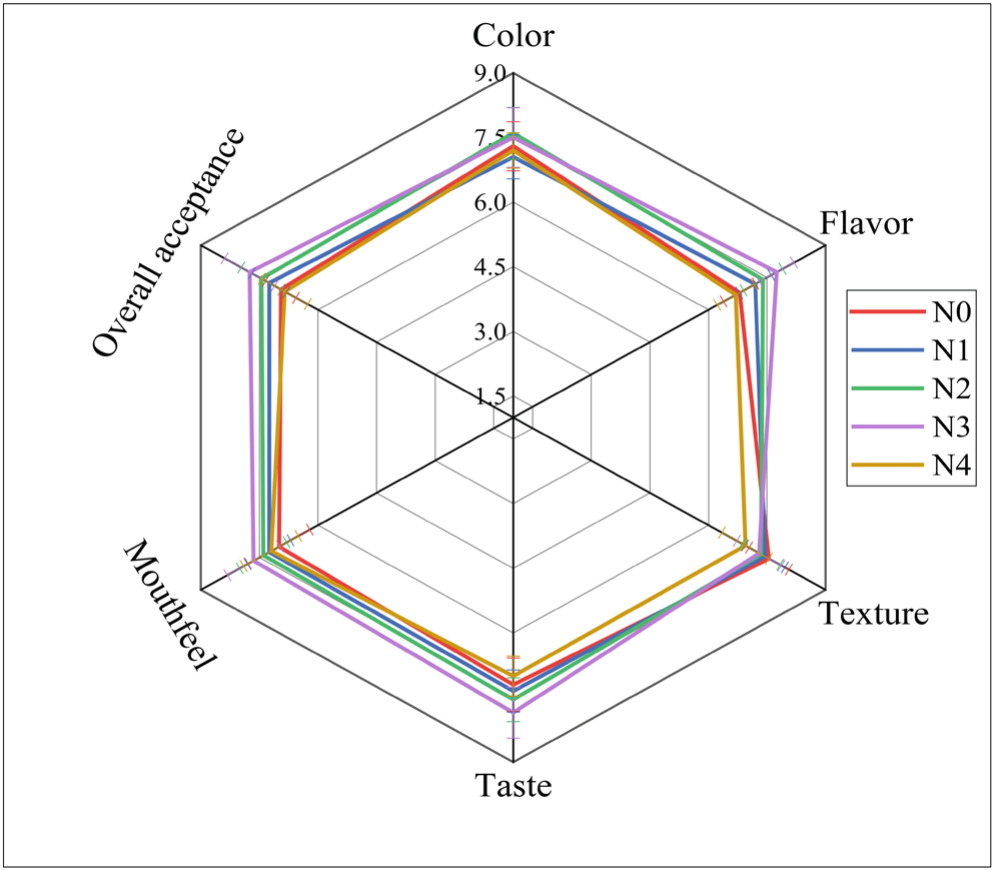
Sensory attributes of noodles. N0 = 100% FMF, N1 = 85% FMF + 5% TP + 10% DSF, N2 = 80% FMF + 10% TP + 10% DSF, N3 = 75% FMF + 15% TP + 10% DSF, N4 = 70% FMF + 20% TP + 10% DSF. Values are means of triplicates ± standard deviation (*n* = 20).

Sensory attributes including color, flavor, texture, taste, mouthfeel, and overall acceptability of noodles are presented in Figure 5. In terms of color, no significant (*p* < 0.05) differences were observed in the scores of experimental noodles compared to N0. However, a numerical rise in the scores was observed after TP and DSF incorporation such as N2 and N3 got higher scores compared to N0. The increase in redness (a*) and yellowness (b*) (as described in Section 3.4) produced a more appealing hue, which corresponded with panelists' higher color preference for N2 and N3. This aligns with the study by (Concha‐Meyer et al. 2019) where the sensory analyses indicated an enhancement in both the visual appeal and color attractiveness of wheat bread at 5% and 10% tomato pomace powder concentrations. However, a decline was noticed in the score of N4, which might be due to excessively saturated color derived from increased a* and b* values (as detailed in Section 3.4), which diminished panelists' preference. In the case of flavor, N2 and N3 scored notably (*p* < 0.05) higher ratings compared to N0. Tomato powder contains several aroma volatiles such as 2‐methylbutanoate, β‐ionone, hexanal, etc., which are associated with its favorable aroma (Jeyaprakash et al. 2020). However, N4 got lower values among all the experimental noodles, which was likely due to its intense flavor derived from the highest proportion of TP (20%). In terms of taste, only N3 got significantly (*p* < 0.05) higher preferences compared to N0. A study by (Solhi et al. 2020) reported that processed cheese samples with tomato powder received higher scores for color, flavor, and overall acceptability compared to the control samples. The scores for texture, in comparison to N0, exhibited no significant (*p* < 0.05) changes except for N4. N4 got significantly (*p* < 0.05) lower scores compared to other TP and DSF enriched noodles (N1–N3) in terms of texture, which might be attributed to the maximum drop in hardness, cohesiveness, springiness, and adhesiveness compared to other experimental samples, consistent with instrumental texture findings (Section 3.5). This suggests that the weakened structural integrity of N4 was perceptible to panelists. Among all the noodles, N3 got substantially (*p* < 0.05) greater score in terms of mouthfeel. Therefore, Figure 5 demonstrates that noodle samples containing 15% TP (N3) was predominantly favored by the panelists in terms of overall acceptance.

## 4 Conclusion

This study demonstrated that integrating tomato powder and defatted soybean flour into gluten‐free noodles substantially enhanced their nutritional profile while maintaining cooking performance and sensory acceptability. These factors are vital for ensuring consumer satisfaction and achieving success in the market. The optimized formulation with 15% TP and 10% DSF appeared to be the most balanced for nutritional enhancement, including antioxidant activity, phenolics, flavonoids, dietary fiber, protein, and minerals and consumer appeal, providing an appealing gluten‐free alternative. These findings emphasize the prospect of integrating plant‐derived bioactive components to develop gluten‐free products that are not only beneficial for health but also palatable. However, future studies should evaluate nutrient bioavailability and postprandial glycemic response of the optimized formulation. Additionally, validating health advantages of the designed noodles via clinical trials, evaluating storage stability, and investigating commercialization prospects should be emphasized in further research.

## Author Contributions

**Abu Tareq Mohammad Abdullah:** writing – review and editing, resources, supervision, visualization. **Tasnim Farzana:** conceptualization, investigation, funding acquisition, project administration, supervision. **Md. Jaynal Abedin:** investigation, writing – original draft, methodology, validation, writing – review and editing, formal analysis, software. **Mitu Rani Sarker:** writing – original draft, methodology, data curation, formal analysis, software, validation.

## Data Availability

The data that support the findings of this study are available from the corresponding author upon reasonable request.
